# Predictive value of preoperative T cell phenotypes for postoperative pulmonary complications in elderly patients undergoing thoracic surgery

**DOI:** 10.1016/j.jatmed.2026.07.002

**Published:** 2026-08-10

**Authors:** Wen Liu, Yuting Liu, Tiantian Zhou, Xingqi Zhao, Jianjun Yang, Qingren Liu, Muhuo Ji

**Affiliations:** a Department of Anesthesiology, The Second Affiliated Hospital of Nanjing Medical University, Nanjing 210000, China; b Department of Anesthesiology, Nanjing Integrated Traditional Chinese and Western Medicine Hospital Affiliated with Nanjing University of Chinese Medicine, Nanjing 210014, China; c Department of Anesthesiology, Pain and Perioperative Medicine, The First Affiliated Hospital of Zhengzhou University, Zhengzhou 450000, China; d Department of Anesthesiology, Xishan People’s Hospital of Wuxi City, Wuxi 214105, China

**Keywords:** T cell subsets, Programmed death protein-1, Postoperative pulmonary complications

## Abstract

**Background:**

Postoperative pulmonary complications (PPCs) significantly impair patient recovery, yet reliable predictors remain scarce. T cell and mitochondrial phenotypic marker abnormalities are recognized as key mechanisms underlying immune dysregulation. Accordingly, we hypothesized that quantifying these T cell characteristics could serve as a novel predictive strategy for PPCs.

**Methods:**

Patients who underwent elective thoracic surgery under general anesthesia were enrolled in this study. Preoperative venous blood samples were collected to measure T cell subsets and their mitochondrial function indicators.

**Results:**

The occurrence of PPCs was evaluated postoperatively. Among the 176 enrolled patients, 44 (25.0%) developed PPCs. Compared with the non-PPCs group, patients in the PPCs group were older, had longer operative time, and exhibited significantly higher preoperative levels of C-reactive protein, neutrophils, white blood cells, prothrombin time, activated partial thromboplastin time, fibrinogen degradation products, D-dimer, and international normalized ratio, while the preoperative lymphocyte percentage was significantly lower (*P* < 0.05). Regarding T cell related parameters, the PPCs group showed a significantly lower absolute count of CD3^+^CD4^+^ T cells, significantly higher percentage and absolute count of CD3^+^CD4^+^PD-1^+^ T cells, and a significantly lower percentage of CD3^+^CD8^+^MMP T cells (*P* < 0.05). Multivariable logistic regression analysis identified the percentage of CD3^+^CD4^+^PD-1^+^ T cells, absolute count of CD3^+^CD4^+^PD-1^+^ T cells, and operative time as independent risk factors for PPCs, while lymphocyte percentage emerged as an independent protective factor. After adjustment using different models, the multivariable model (Model 3) demonstrated that both the percentage and absolute count of CD3^+^CD4^+^PD-1^+^ T cells had good predictive performance for PPCs (*P* < 0.001).

**Conclusions:**

Thus, preoperative measurement of T cell subsets indicators may aid in early identification and risk stratification of PPCs after thoracic surgery.

## Introduction

Postoperative pulmonary complications (PPCs), a common occurrence following major surgeries, include atelectasis, pneumonia, acute exacerbation of chronic obstructive pulmonary disease (AECOPD), acute lung injury (ALI), acute respiratory distress syndrome (ARDS), and respiratory failure.^1^ The reported incidence of PPCs is high, ranging from 12% to 50%. These complications are associated with prolonged hospital stays, increased morbidity, significant disability rates, and substantial healthcare expenditures.^2^, ^3^ Unfortuantely, robust and reliable predictive tools for PPCs remain to be investigated. Thus, identification of risk factors associated with blood-based biomarkers is crucial for the early detection of PPCs.

T cells are central components of the human immune system. T cell dysfunction denotes a state of immune dysfunction, characteristically marked by elevated expression of programmed cell death protein 1 (PD‐1)^4^. PD‐1 upregulation is a hallmark shared by both chronic infections and cancer.^5^ Increased PD‐1 expression on dysfunctional T cells has been significantly associated with a higher incidence of pulmonary fungal infections.^6^ Consequently, this T cell dysfunction compromises pathogen clearance and weakens the control of inflammatory responses. Surgical stress and tissue injury can further trigger systemic immune dysregulation, marked by elevated lymphocyte apoptosis, especially among T cell subsets. As a result, postoperative inflammation may be prolonged, further aggravating lung tissue injury.^6^, ^7^

Mitochondrial dysfunction is primarily characterized by reduced mitochondrial membrane potential (MMP) and altered mitochondrial mass (MM).^8^ The postoperative hypermetabolic state and inflammatory microenvironment can significantly impair mitochondrial function in immune cells, adversely affecting their bioenergetic metabolism, dynamic plasticity, and signaling pathways.^9^ Critically, an abnormally reduced MMP may fail to provide adequate energy for effective immune responses or to ensure proper T cell proliferation and differentiation, thereby exacerbating immunosuppression. This adaptive immune impairment likely reduces pathogen clearance and delays tissue repair, establishing a critical biological foundation for the development and progression of PPCs.^10^

Therefore, we hypothesize that aberrant PD‐1 expression and mitochondrial phenotypic markers in T cells may offer new insights into predicting PPC risk in patients undergoing thoracic surgery. This study aimed to investigate the associations between preoperative PD‐1 expression on T cell subsets, their mitochondrial phenotypic markers indicators, and the occurrence of PPCs, thereby providing a reference for clinical perioperative risk assessment.

## Methods

### Recruitment of patients

A total of 176 patients who underwent elective thoracic surgery under general anesthesia at the Second Affiliated Hospital of Nanjing Medical University between January 2024 and November 2024 were enrolled in this study. Inclusion criteria were as follows: (1) age ≥ 60 years, with voluntary participation and signed informed consent; (2) availability of complete clinical data and ability to complete follow-up visits; (3) scheduled for thoracic surgery; and (4) American Society of Anesthesiologists (ASA) physical status class I–III. Exclusion criteria included: (1) inability to communicate due to coma, severe dementia, or language barriers; (2) history of drug or alcohol abuse or significant psychiatric illness; (3) presence of severe cardiac, renal, hepatic, gastrointestinal, infectious, endocrine, or malignant diseases; and (4) Preoperative use of immune checkpoint inhibitors. All patients completed relevant preoperative examinations during hospitalization. None of the included patients were diagnosed with infection prior to surgery.

### Ethical approval and registration

This study was reviewed and approved by the Ethics Committee of the Second Affiliated Hospital of Nanjing Medical University, Jiangsu Province, China (Approval No. 2024‐KY‐174‐02). The trial was registered in the Chinese Clinical Trial Registry (Registration No. ChiCTR2400087577). Written informed consent has been obtained from the participants to publish this paper.

### Study design

This study was designed as a prospective case-control investigation. All enrolled patients completed a standard preoperative assessment, including evaluations for T cell and mitochondrial phenotypic markers conducted on the day before surgery. Postoperative follow-up was performed daily for 7 consecutive days by experienced members of the research team blinded to the preoperative laboratory results (including T cell and mitochondrial phenotypic markers assessments). They could voluntarily withdraw from the study at any time and for any reason without impact on subsequent clinical care. Preoperative evaluations were performed on the day before surgery. The following data were collected: age, sex, educational level, body mass index (BMI), preoperative comorbidities (hypertension, diabetes mellitus, cerebral infarction), duration of surgery, American Society of Anesthesiologists (ASA) physical status classification, complete blood count, biochemical profiles, coagulation function, and results of T cell and mitochondrial phenotypic markers testing.

### Anesthesia protocol

No patient received preoperative medication, and routine fasting protocols were followed. Upon entering the operating room, peripheral venous access was established. Standard monitoring included electrocardiography (ECG), heart rate (HR), non‑invasive blood pressure (NIBP), bispectral index (BIS), and peripheral oxygen saturation (SpO₂). Invasive arterial pressure (IBP) monitoring was also applied. Oxygen was administered via a face mask at 3–5 L/min. Anesthesia was induced intravenously with sufentanil (0.4 μg/kg), propofol (2.02.5 mg/kg), and rocuronium (0.6 mg/kg). Maintenance consisted of sevoflurane (1.0%−2.0%) inhalation, with continuous intravenous infusion of propofol (3.0–6.0 mg·kg⁻¹·h⁻¹) and remifentanil (0.05–0.2 μg·kg⁻¹·min⁻¹). Muscle relaxation was maintained with intermittent boluses of rocuronium, and the BIS value was kept between 40 and 60. A double‑lumen endobronchial tube was placed under bronchoscopic guidance. Its position was reconfirmed by fiberoptic bronchoscopy and auscultation after positioning the patient in the lateral decubitus position. During two-lung ventilation, a protective strategy was employed with a tidal volume of 6–8 mL/kg, PEEP, and recruitment maneuvers. Before one-lung ventilation, 100% FiO₂ was given to expedite lung collapse. During one‑lung ventilation, tidal volume was set to 4–6 mL/kg with PEEP 5–10 cmH₂O while maintaining PETCO₂ at 35–45 mmHg. Peak and plateau pressures were limited to < 30 cmH₂O and < 20 cmH₂O, respectively. For analgesia, all patients received a paravertebral nerve block intraoperatively. Postoperatively, a patient-controlled intravenous analgesia pump was provided, delivering sufentanil at a background rate of 2 μg/h, with a bolus dose of 2 μg and a lock-out interval of 10 min, to minimize systemic opioid consumption while ensuring adequate pain control.

### Detection of T cell and mitochondrial phenotypic markers

Peripheral blood samples were collected from patients in anticoagulant tubes. Peripheral blood mononuclear cells were isolated by density gradient centrifugation, followed by washing 2–3 times with buffer to remove impurities and to adjust the cell concentration for flow cytometry. For staining, the cell suspension was aliquoted into flow cytometry tubes and incubated for 15–30 min in the dark with fluorescently labeled antibodies against T cell subsets (CD3, CD4, CD8, CD45), the exhaustion marker PD‐1, and mitochondrial probes (MMP‑sensitive dye and mitochondrial mass detection reagents). Unbound antibodies were removed by washing with buffer. Flow cytometry was performed after instrument calibration and setup of positive/negative controls. The stained cells were analyzed by detecting fluorescence and light scatter signals, with target cell populations gated for data acquisition. Data were then analyzed using flow cytometry software. The relative percentage and absolute count (cells/μL) of each population were calculated. Results were subsequently compared with reference ranges to identify abnormalities. Throughout the procedure, temperature, incubation time, and light protection were strictly controlled to ensure accuracy.

### Outcome measures

PPCs refer to newly developed pulmonary conditions occurring during the postoperative hospital stay. These primarily include pneumonia, respiratory failure, pleural effusion, atelectasis, pneumothorax, bronchospasm, and aspiration pneumonia. Notably, pleural effusion or pneumothorax was considered a PPC only when it occurred in the non‑operative lung. Diagnostic criteria were based on the 2015 European Perioperative Clinical Outcomes definitions:^11^, ^12^ (1) Pneumonia: Radiographic evidence (infiltration, consolidation, or cavitation) plus at least one systemic sign (body temperature >38 °C without other cause; WBC < 4 or >12 × 10⁹/L; or altered mental status in patients >70 years) plus at least two respiratory findings (new/worsening purulent sputum or change in character, increased secretions or suction volume, new/worsening cough or dyspnea/tachypnea, rales or bronchial breath sounds, or impaired gas exchange). (2) Respiratory failure: PaO₂ < 60 mmHg on room air, PaO₂/FiO₂ < 300 mmHg, or new requirement for oxygen therapy due to hypoxemia. (3) Pleural effusion: Chest X‑ray showing blunting of the costophrenic angle, loss of the diaphragmatic contour, displacement of adjacent structures, or lung opacification with preserved vascular markings in the supine position. (4) Atelectasis: Pulmonary opacification with associated mediastinal shift, hilar/diaphragmatic displacement toward the affected area, or compensatory hyperinflation of adjacent lung. (5) Pneumothorax: Radiographic evidence of air in the pleural space with absent vascular markings peripheral to the visceral pleural line. (6) Bronchospasm: New onset of expiratory wheezing requiring bronchodilator treatment. (7) Aspiration Pneumonia: Acute lung injury resulting from aspiration of gastric contents.

### Statistical analysis

Using PPCs as the binary outcome, this study employed multivariable logistic regression and ROC curves to assess the predictive value of preoperative T cell subsets and their mitochondrial function parameters for PPCs. Given the exploratory nature of this prediction study, sample size adequacy was evaluated based on the events-per-variable (EPV) principle, which recommends approximately 10 outcome events per candidate variable in logistic regression to minimize the risk of overfitting and ensure estimation stability. A total of 176 elderly patients undergoing thoracic surgery were enrolled, of whom 44 (25.0%) developed PPCs. According to the 10 EPV rule, the current sample size theoretically supports the inclusion of approximately four degrees of freedom of core predictors in the final model. Using stepwise regression combined with clinical judgment, the final model primarily included the percentage (or absolute count) of CD3^+^CD4^+^PD‐1^+^ T cells, lymphocyte percentage, operative time, and a few other key variables. Thus, the sample size is generally adequate for exploratory multivariable analysis. ROC curves were further used to evaluate the predictive performance of individual indicators and their combined models. Nevertheless, given the single-center design and the relatively limited sample size and number of events, these findings should be considered preliminary. External validation in larger, independent cohorts is warranted to confirm their robustness and generalizability.

Statistical analysis was performed using SPSS 26.0 and GraphPad Prism software. A stratified strategy was applied to handle missing data: variables with > 20% missing values were excluded from the analysis; whereas those with ≤ 20% missingness were handled via multiple imputation using the Multiple Imputation by Chained Equations (MICE) method. Normally distributed continuous data are presented as mean ± standard deviation and compared using the independent samples *t*-test. Non‑normally distributed continuous data are expressed as median (interquartile range, IQR) and analyzed with the Mann-Whitney *U* test. Categorical and ordinal variables are summarized as counts (percentages) and compared using the chi‑square test or Fisher’s exact test, as appropriate.Variables with statistical significance in univariate analysis were entered into a binary logistic regression model to identify independent risk factors for PPCs. A two-tailed *P* < 0.05 was considered statistically significant. Results were presented as odds ratios (OR) with 95% confidence intervals (CI). Three stepwise-adjusted regression models were constructed: Model 1 was a crude model without adjustment for covariates; Model 2 was adjusted for age and operative time; Model 3 was further adjusted for age, operative time, lymphocyte percentage, D-Dimer and CD3^+^CD8^+^MMP T cell percentage. Model 3 performance was assessed by discrimination (AUC and Brier score) and calibration (Hosmer-Lemeshow test and calibration plot). To further evaluate the predictive performance of the independent PPCs-related indicators identified by binary logistic regression analysis, two combined prediction models were established. Combined test 1 included the percentage of CD3^+^CD4^+^PD‐1^+^ T cells and operative time; Combined test 2 included the absolute count of CD3^+^CD4^+^PD‐1^+^ T cells, operative time, and lymphocyte percentage. The predictive performance of the two combined models was compared to explore the incremental value of immune-related indicators in PPCs risk prediction. Receiver operating characteristic (ROC) curves were generated using MedCalc software to calculate the area under the ROC curve (AUC), sensitivity, and specificity for each factor. Nomogram construction was performed using R software (version 4.5.1) with the rms package. Based on the results of the multivariate logistic regression analysis, the lrm function was used to fit the model, and the nomogram function was employed to generate a nomogram, which intuitively illustrated the contribution of each risk factor to the predicted probability of PPCs.

Restricted cubic spline (RCS) curves were fitted using the rms package in R software, and data visualization was performed using the ggplot2 package, generating curves for the percentage and absolute count of CD3^+^CD4^+^PD‐1^+^ T cells in Model 3.

## Results

In this study, among the 176 patients included in the analysis, 44 (25.0%) developed PPCs postoperatively. The patient selection process is summarized in the study flowchart (Fig. S1). Compared with the non-PPCs group, patients in the PPCs group were significantly older and had a longer operative time (*P* < 0.05). Preoperative laboratory findings revealed that, compared with the non-PPCs group, the PPCs group had significantly higher levels of C-reactive protein, neutrophils, white blood cells, prothrombin time, activated partial thromboplastin time, fibrinogen degradation products, D-dimer, and international normalized ratio (INR) (*P* < 0.05), whereas lymphocyte percentage and albumin levels were significantly lower (*P* < 0.05). No significant differences were observed between the two groups in terms of ASA classification, BMI, or preoperative comorbidities (hypertension, diabetes mellitus, coronary artery disease) (*P* > 0.05; Table 1).

**Table 1:** Comparison of general characteristics between the two groups.

**Variable**	**non-PPCs**	**PPCs**	***P*****-value**
	**n = 132**	**n = 44**	
Male/Female (n)	69/63	28/16	0.189
Age (years)	68.00 (63.50, 73.00)	72.00 (65.00, 76.00)	0.019
BMI (kg/m^2^)	23.44 ± 2.85	22.91 ± 3.30	0.308
Urban/Rural (n)	90/42	27/17	0.388
ASA Ⅱ/Ⅲ (n)	76/54	21/22	0.530
Smoking History [n (%)]			0.084
No	101 (77)	39 (89)	
Yes	31 (23)	5 (11)	
Drinking History [n (%)]			0.612
No	113 (86)	39 (89)	
Yes	19 (14)	5 (11)	
Surgical History [n (%)]			0.138
No	67 (51)	28 (64)	
Yes	65 (49)	16 (36)	
Comorbid diseases [n (%)]			
Hypertension	59 (45)	23 (52)	0.383
Diabetes Mellitus	20 (15)	7 (16)	0.904
Coronary Heart Disease	10 (8)	4 (9)	0.758
Postoperative Diagnosis of Malignant Tumor [n (%)]			0.213
No	22 (18)	9 (28)	
Yes	99 (82)	23 (72)	
Operation Time (min)	110.00 (90.00, 160.00)	145.00 (100.00, 285.00)	0.012
Blood Loss (mL)	50.00 (20.00, 85.00)	50.00 (30.00, 100.00)	0.125
Pre Neutrophil Percentage (%)	56.69 ± 10.10	59.76 ± 9.64	0.080
Pre Neutrophil Count (10^9^/L)	2.85 (2.25, 4.04)	3.15 (2.56, 3.90)	0.011
Pre Lym Count (10^9^/L)	1.59 (1.27, 1.94)	1.52 (1.12, 2.00)	0.698
Pre Lym Percentage (%)	31.82 ± 9.65	28.22 ± 9.36	0.032
Pre WBC (10^9^/L)	5.07 (4.32, 6.25)	5.61 (4.61, 6.35)	0.019
Pre CRP (mg/L)	0.40 (0.40, 1.83)	0.82 (0.40, 4.10)	0.015
Pre ALB (g/L)	42.49 ± 3.88	41.32 ± 5.03	0.112
Pre PT (s)	12.30 (11.80, 12.80)	12.60 (12.05, 13.25)	0.010
Pre APTT (s)	31.50 (29.40, 34.40)	33.40 (31.75, 37.10)	0.003
Pre FDP (μg/mL)	2.80 (2.10, 3.50)	3.10 (2.70, 3.95)	0.004
Pre D-Dimer (μg/mL)	0.67 (0.56, 0.90)	0.84 (0.67, 1.29)	0.002
Pre INR	0.95 (0.90, 0.99)	0.99 (0.95, 1.02)	0.004

Data are presented as n (%), mean ± standard deviation, or median (interquartile range), depending on variable type and distribution. ASA, American Society of Anesthesiologists; BMI, body mass index; Lym, lymphocyte; WBC, white blood cell; CRP, C-reactive Protein; ALB, Albumin; PT, Prothrombin Time; APTT, Activated Partial Thromboplastin Time; FDP, Fibrinogen Degradation Products; INR, International Normalized Ratio.

**Fig. 1:**
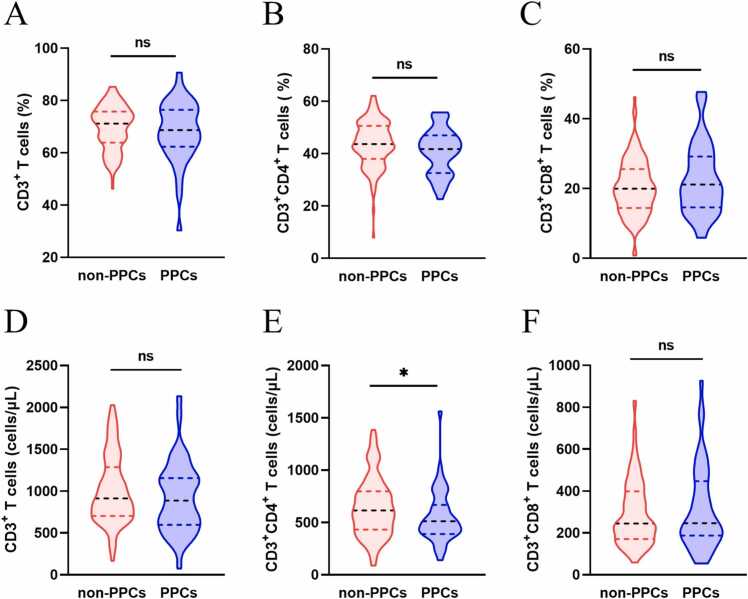
Comparison of T cell subsets between the two groups. Note: %, percentage. *P* < 0.05 was considered statistically significant. *P < 0.05.

As shown in Table S1 and Fig. 1, T cell subset analysis revealed that the absolute count of CD3^+^CD4^+^ T cells was significantly lower in the PPCs group compared with the non-PPCs group (*P* < 0.05). No significant differences were observed in the remaining parameters (*P* > 0.05).

**Fig. 2:**
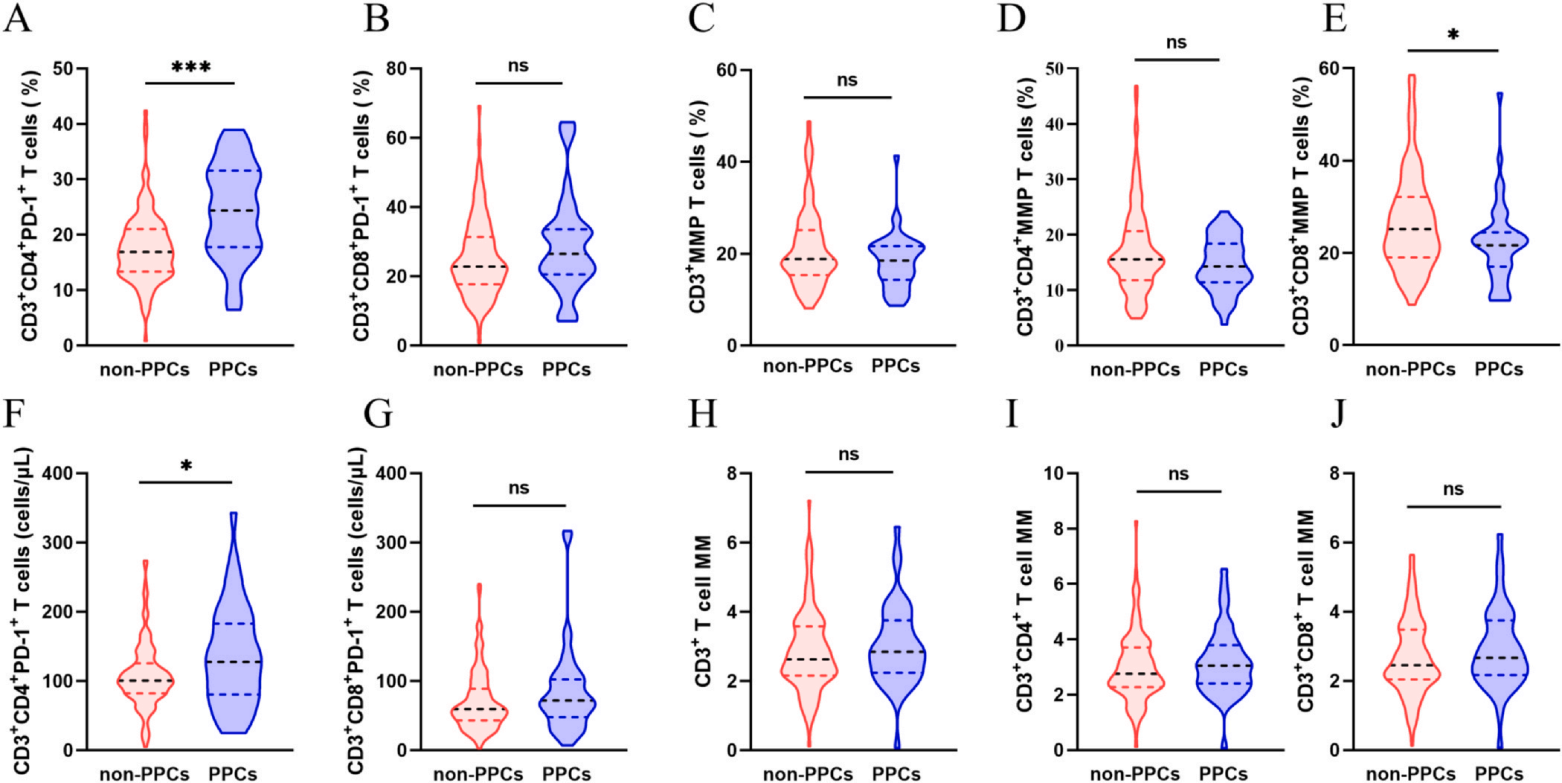
Comparison of PD‐1 expression and mitochondrial function-related indicators in T cell subsets between the two groups. Note: PD‐1, programmed cell death protein‐1; MMP, mitochondrial membrane potential; MM, mitochondrial mass; PPCs, postoperative pulmonary complications; %, percentage. *P* < 0.05 was considered statistically significant. *P < 0.05; ***P < 0.001.

In this study, the percentage and absolute count of CD3^+^CD4^+^PD‐1^+^ T cells were significantly higher in the PPCs group than in the non-PPCs group (*P* < 0.05), with no significant differences reported in the remaining indicators (Table S2-1 and Fig. 2). Additionally, the percentage of CD3^+^CD8^+^MMP T cells was significantly lower in the PPCs group compared with the non-PPCs group (*P* < 0.05), whereas no significant differences were found in the other indicators (Table S2-2 and Fig. 2).

Variables with *P* < 0.05 were entered into a binary logistic regression model. Collinearity diagnostics revealed that neutrophils and the percentage of CD3^+^CD4^+^ T cells had variance inflation factor (VIF) values exceeding 10, indicating severe multicollinearity; therefore, these variables were excluded. Univariate logistic regression results are presented in Table S3-1. The remaining variables were included in the multivariable analysis. To avoid collinearity interference, two separate multivariable logistic regression models were constructed: one incorporating the percentage of CD3^+^CD4^+^PD‐1^+^ T cells, and the other incorporating the absolute count of CD3^+^CD4^+^PD‐1^+^ T cells. The results showed that the percentage of CD3^+^CD4^+^PD‐1^+^ T cells (OR = 1.140, 95% CI: 1.074–1.210, *P* < 0.001), the absolute count of CD3^+^CD4^+^PD‐1^+^ T cells (OR = 1.020, 95% CI: 1.011–1.030, *P* < 0.001), and operative time (OR = 1.008, 95% CI: 1.004–1.013, *P* = 0.001) were independent risk factors for PPCs, whereas lymphocyte percentage (OR = 0.926, 95% CI: 0.873–0.982, *P* = 0.010) was an independent protective factor (Table S3-2 and Table S3-3). Multivariable binary logistic regression analysis was further performed to assess potential independent influencing factors of PPCs. After adjusting for age and operative time, both the percentage of CD3^+^CD4^+^PD‐1^+^ T cells (OR = 1.151, 95% CI: 1.086–1.219, *P* < 0.001) and the absolute count of CD3^+^CD4^+^PD‐1^+^ T cells (OR = 1.012, 95% CI: 1.005–1.018, *P* = 0.001) remained significantly associated with PPCs (Table 2, Model 2). After further adjustment for five covariates based on univariate logistic regression results with *P* < 0.05, namely age, operative time, lymphocyte percentage, D-Dimer and CD3^+^CD8^+^MMP T cell percentage, both the percentage of CD3^+^CD4^+^PD‐1^+^ T cells (OR = 1.137, 95% CI: 1.072–1.205, *P* < 0.001) and the absolute count of CD3^+^CD4^+^PD‐1^+^ T cells (OR = 1.013, 95% CI: 1.006–1.021, *P* < 0.001) remained significantly associated with the occurrence of PPCs (Table 2, Model 3). For brevity, Table 2 presents only the core immunological predictors of interest; the full multivariable models with all covariates are provided in Table S4-1 and Table S4-2. Internal validation of Model 3 was performed by assessing discrimination and calibration. The model incorporating CD3^+^CD4^+^PD‐1^+^ T cell percentage demonstrated good discriminative ability (AUC = 0.815, Brier score = 0.137), with adequate calibration (Hosmer-Lemeshow χ² = 7.605, df = 8, *P* = 0.473). Similarly, the model incorporating CD3^+^CD4^+^PD‐1^+^ T cell absolute count also showed acceptable performance (AUC = 0.777, Brier score = 0.137) and adequate calibration (Hosmer-Lemeshow χ² = 3.497, df = 8, *P* = 0.899; Table S8). Calibration plots for both models showed good agreement between predicted probabilities and observed event rates (Fig. S4-1 and Fig. S4-2).

**Table 2:** Multivariable logistic regression analysis for PPCs.

	**Model 1**		**Model 2**		**Model 3**	
	**OR (95% CI)**	***P*****-value**	**OR (95% CI)**	***P*****-value**	**OR (95% CI)**	***P*****-value**
CD3^+^CD4^+^PD‐1^+^ T cell percentage	1.132 (1.075–1.193)	<0.001	1.151 (1.086–1.219)	<0.001	1.137 (1.072–1.205)	<0.001
CD3^+^CD4^+^PD‐1^+^ T cell count	1.009 (1.003–1.015)	0.006	1.012 (1.005–1.018)	0.001	1.013 (1.006–1.021)	<0.001

In Model 1, the percentage and absolute count of CD3^+^CD4^+^PD‐1^+^ T cells were not adjusted. In Model 2, the percentage and absolute count of CD3^+^CD4^+^PD‐1^+^ T cells were adjusted for age and operative time, respectively. In Model 3, the percentage and absolute count of CD3^+^CD4^+^PD‐1^+^ T cells were adjusted for age, operative time, lymphocyte percentage, D-Dimer and CD3^+^CD8^+^MMP T cell percentage. PD‐1, programmed cell death protein‐1; OR, odds ratio; CI, confidence interval. *P* < 0.05 was considered statistically significant.

**Fig. 3:**
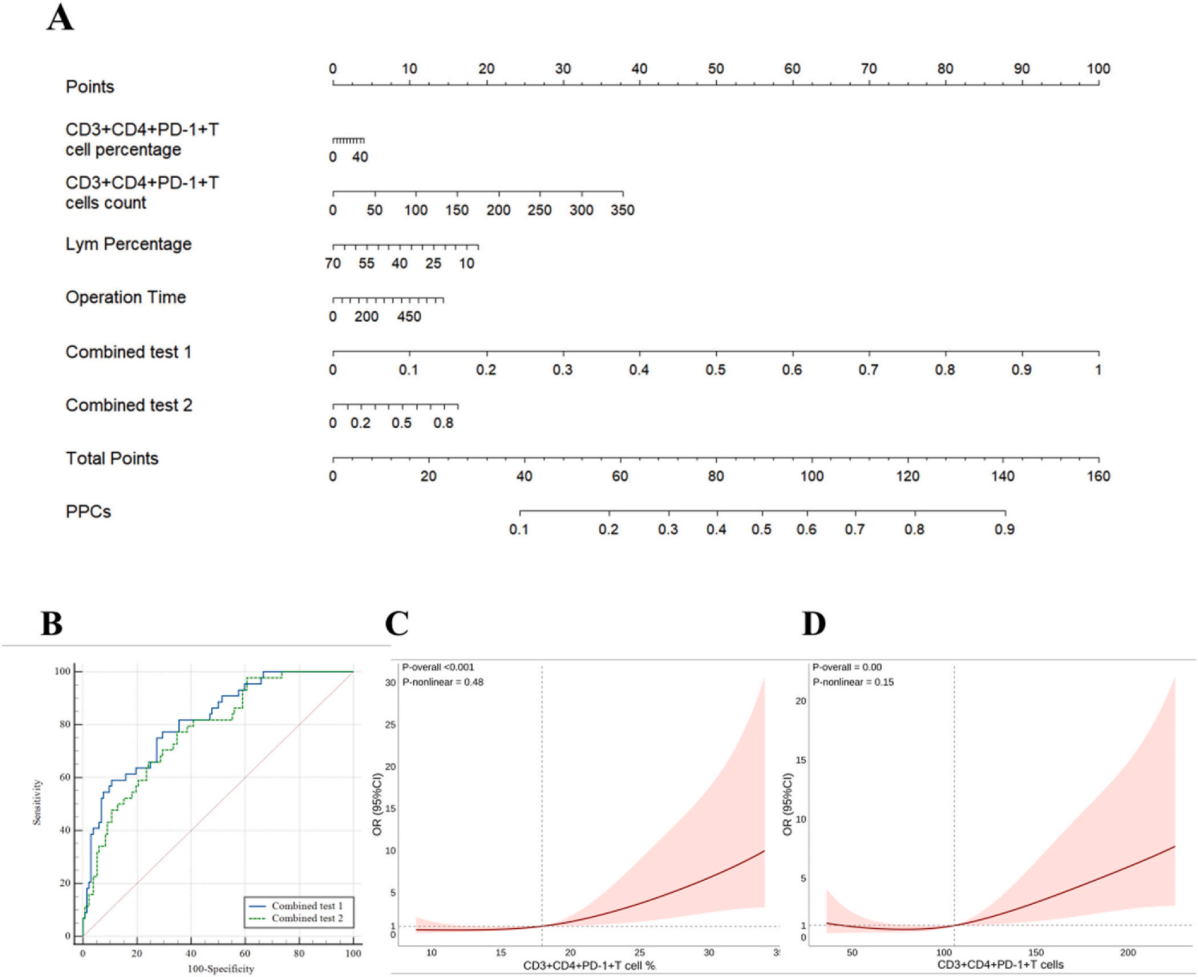
(A) Nomogram for individualized PPC risk prediction; (B) ROC curves of combined tests for predicting PPCs; (C) RCS plot of the relationship between CD3^+^CD4^+^PD‐1^+^ T cell percentage and PPCs; (D) RCS plot of the relationship between absolute count of CD3^+^CD4^+^PD‐1^+^ T cells and PPCs. Note: Combined test 1: combination of CD3^+^CD4^+^PD‐1^+^ T cell percentage and operative time for predicting PPCs; Combined test 2: combination of absolute count of CD3^+^CD4^+^PD‐1^+^ T cells, operative time, and lymphocyte percentage for predicting PPCs; Both panels C and D are adjusted based on multivariable logistic regression Model 3. CI, confidence interval; %, percentage.

To evaluate the predictive ability of these indicators for PPCs, ROC curves were plotted with patients who developed PPCs as the positive sample and those who did not as the negative sample, and their characteristics were summarized (Table S5-1 and Table S5-2). The results showed that the AUC of CD3^+^CD4^+^PD‐1^+^ T cell percentage for predicting PPCs was 0.741 (95% CI: 0.649–0.834, *P* < 0.001), the AUC of absolute count of CD3^+^CD4^+^PD‐1^+^ T cells was 0.610 (95% CI: 0.500–0.721, *P* = 0.029), and the AUC of operative time was 0.627 (95% CI: 0.529–0.725, *P* = 0.012). The combination of CD3^+^CD4^+^PD‐1^+^ T cell percentage and operative time (Combined test 1) yielded an AUC of 0.815 (95% CI: 0.749–0.869, *P* < 0.001), with a sensitivity of 59.1% and a specificity of 89.4% (Table S5-1 and Fig. 3B). The combination of absolute count of CD3^+^CD4^+^PD−1^+^ T cells, operative time, and lymphocyte percentage (Combined test 2) yielded an AUC of 0.777 (95% CI: 0.708–0.836, *P* < 0.001), with a sensitivity of 77.3% and a specificity of 65.2% (Table S5-2 and Fig. 3B). Based on the independent predictive factors identified above, we constructed a nomogram to provide a more intuitive tool for estimating the individual risk of PPCs in elderly patients (Fig. 3A).

Spearman correlation analysis revealed a moderate positive correlation between the percentage and absolute count of CD3^+^CD4^+^PD‐1^+^ T cells (ρ = 0.387, *P* < 0.001; Table S7-1). Notably, these two measures showed divergent correlation patterns with other clinical variables: percentage was negatively correlated with lymphocyte percentage (ρ = −0.202, *P* = 0.007) and CD3^+^CD8^+^MMP T cell percentage (ρ = −0.264, *P* < 0.001), whereas absolute count was positively correlated with lymphocyte percentage (ρ = 0.264, *P* < 0.001) and WBC (ρ = 0.394, *P* < 0.001), supporting their complementary rather than redundant roles (Table S7-2 and Table S7-3).

To further evaluate whether the predictive value of these T cell parameters is modified by operative duration, we conducted stratified subgroup analyses. Subgroup analysis based on median operative time (115 min) showed that the association between CD3^+^CD4^+^PD‐1^+^ T cell percentage and PPCs was consistent across strata, with no significant interaction (*P* for interaction = 0.129). The OR was 1.21 (95% CI: 1.09–1.34, *P* < 0.001) for operative time < 115 min and 1.11 (95% CI: 1.04–1.18, *P* = 0.001) for operative time ≥ 115 min (Fig. S2). The positive association between absolute count CD3^+^CD4^+^PD‐1^+^ T cells and the risk of PPCs was consistent across both operative time strata. For patients with operative time < 115 min, the OR was 1.01 (95% CI: 1.00–1.02, *P* = 0.015); for those with operative time ≥ 115 min, the OR was 1.01 (95% CI: 1.00–1.02, *P* = 0.011). The test for interaction yielded a *P* value of 0.907, indicating no significant effect modification by operative time (Fig. S3).

To explore whether CD3 +CD4 +PD‐1 + T cell parameters are linearly associated with PPCs, we constructed dose-response curves using restricted cubic spline (RCS) with adjustment for all covariates in Model 3. The red curves depict the fitted relationship between these T cell parameters and the probability of PPCs, with light red shaded areas representing the 95% confidence intervals. The results indicate that both the percentage (Fig. 3C) and absolute count (Fig. 3D) of CD3^+^CD4^+^PD‐1^+^ T cells exhibit a linear relationship with the probability of PPCs: higher levels of this T cell subset are associated with an increased risk of PPCs. This linear dose‑response relationship was further confirmed by sensitivity analyses using quadratic terms, which were not significant for either the percentage model (*P* = 0.890) or the absolute count model (*P* = 0.430; Table S6-1 and Table S6-2). Collectively, these findings support that CD3^+^CD4^+^PD‐1^+^ T cells are an independent risk factor for PPCs with a linear dose‑response pattern.

## Discussion

In this observational study, we comprehensively investigated the associations of PD‐1 expression in T cell subsets and MMP related indicators with the occurrence of PPCs. The incidence of PPCs in our thoracic surgery cohort was 25%. Preoperative T cell PD‐1 expression levels and MMP related indicators were significantly associated with PPC occurrence. After adjustment for confounding factors, the percentage and absolute count of CD3^+^CD4^+^PD‐1^+^ T cells, operative time, and lymphocyte percentage remained independently associated with PPCs.

PPCs significantly delay recovery, prolong hospital stays, and increase the risk of secondary issues such as pulmonary infection and respiratory failure. Currently, the preoperative risk assessment for PPCs in thoracic surgery patients primarily relies on traditional clinical indicators, including age, comorbidities, tumor stage, and surgical approach.^13^ These parameters mark an important step in perioperative assessment, yet translating general status evaluation into precise, individualized risk prediction remains an area for continued methodological development. Emerging evidence emphasizes the close link between PPCs and immune-metabolic disturbances, particularly T cell dysfunction and mitochondrial impairment. Mitochondrial activity is critical for sustaining T cell function, and its impairment compromises T cell responses, thereby increasing both the susceptibility to and severity of.^14^

In our study, the most powerful preoperative predictor was an elevated percentage of CD3⁺CD4⁺PD‑1⁺ T cells. In oncology, persistent antigen exposure is known to upregulate PD‑1 on CD4⁺ T cells, driving them toward an exhausted phenotype.^15^, ^16^ As a co‑inhibitory receptor, PD‑1 levels directly reflect T cell functional status.^17^ Under physiological conditions, PD‑1 helps restrain excessive immune activation. However, within the inflammatory milieu of the lung following surgery, sustained pathogen presence and high levels of cytokines such as IL‑6 and TNF‑α can markedly increase PD‑1 expression, leading to T cell dysfunction and compromising the pulmonary immune barrier.^18^ Previous studies have reported a pronounced rise in PD‑1 on T cells after lung resection.^19^, ^20^ Our findings suggest that pre-existing T cell and mitochondrial phenotypic marker abnormalities represents a key vulnerability in surgical patients. Individuals with a higher preoperative burden of T cell phenotypic marker abnormalities may be poorly equipped to mount an effective immune response to surgical stress, increasing susceptibility to postoperative pneumonia, unresolved lung inflammation, atelectasis, and ARDS.^21^

Multivariable logistic regression analysis showed that both the percentage and absolute count of CD3^+^CD4^+^PD-1^+^ T cells were independent predictive risk factors for PPCs. After adjusting for various confounding factors including age and operative time, the associations remained stable for both indicators (Model 3: OR = 1.137, *P* < 0.001; OR = 1.013, *P* < 0.001), suggesting their good predictive value for PPCs. To further improve the predictive performance for PPCs, two combined prediction models were constructed. The results showed that the combination of CD3^+^CD4^+^PD‐1^+^ T cell percentage and operative time (Combined test 1) achieved an AUC of 0.815 for predicting PPCs, which was higher than that of either single indicator. The combination of CD3^+^CD4^+^PD‐1^+^ T cell absolute count, operative time, and lymphocyte percentage (Combined test 2) yielded an AUC of 0.777. These findings indicate that combining immunological indicators with clinical factors can significantly enhance the predictive ability for PPCs.

In this study, RCS curves were used to explore the relationship between CD3^+^CD4^+^PD‐1^+^ T cell parameters (percentage and absolute count) and the risk of PPCs, with adjustment for all covariates in Model 3. The results revealed a linear dose-response relationship, indicating that higher levels of both parameters were significantly associated with an increased risk of PPCs (Fig. 3C,D). The fitted curves suggest that no clear threshold or plateau exists within the observed range; instead, the risk increases progressively with rising PD‐1 expression.^22^, ^23^ This linear pattern may reflect a gradual decline in immune functional reserve as PD‐1 expression increases, without an obvious compensatory phase. In contrast to a nonlinear threshold model, our findings indicate that even modest elevations in PD‐1 expression are associated with a detectable increase in PPCs risk, and that risk continues to rise across the entire spectrum of PD‐1 levels.^24^, ^25^, ^26^ It should be emphasized that this linear relationship, derived from RCS analysis, provides a more robust statistical representation than previously reported smooth curve fitting. The underlying biological mechanisms and clinical generalizability warrant further validation in independent, large-scale cohorts.

MMP serves as a critical foundation for efficient oxidative phosphorylation, and its reduction signifies impaired energy production, mitochondrial dysfunction, and insufficient ATP synthesis. The ensuing oxidative stress can damage cellular lipids, proteins, and DNA, thereby exacerbating tissue injury.^27^, ^28^ Furthermore, it can activate inflammatory pathways and promote the release of pro-inflammatory cytokines, amplifying systemic inflammatory responses.^29^ Ischemia, hypoxia, and oxidative stress resulting from surgical trauma can directly compromise the integrity of the T cell mitochondrial membrane. This leads to decreased MMP and subsequent T cell immune dysfunction. Increased mitochondria-derived ROS can stabilize the HIF1α pathway, upregulating immunosuppressive molecules and reducing cytokine secretion capacity. Consequently, T cells with impaired mitochondrial function are unable to effectively respond to postoperative antigen stimulation and co-stimulatory signals, establishing a close link between metabolic insufficiency and T cell immune dysfunction. Chronic antigen exposure not only upregulates inhibitory receptors such as PD‐1 but also induces a hypometabolic state in T cells.^30^ MM reflects the effective protein content of the respiratory chain in the inner mitochondrial membrane and represents the reserve capacity of cellular metabolism. In the present study, no significant intergroup difference was reported in MM during univariate analysis, nor was any difference found for MMP after multivariate regression adjustment. Nevertheless, the failure of MMP to emerge as an independent predictor warrants further consideration. First, age-related mitochondrial decline in our elderly cohort may have compressed baseline MMP variability, thereby masking its predictive contribution.^31^ Second, mitochondrial dysfunction may represent a downstream consequence of the perioperative inflammatory milieu rather than an independent driver. Surgical trauma-induced ROS have been shown to damage T cell mitochondria and reduce MMP. In this scenario, MMP primarily reflects the cumulative burden of inflammation, offering little additional predictive information beyond upstream markers such as PD‐1 expression.^32^ This may be attributable to the multifactorial nature of PPCs, which are influenced by the extent of surgical trauma, baseline pulmonary function, and postoperative inflammatory responses, suggesting that different immune indicators may have differential predictive value for PPCs.

T cell immunosuppression and mitochondrial metabolic abnormalities are not independent phenomena; rather, their synergistic effect serves as a key driver of PPC development and progression, forming a well-defined causal relationship and a vicious cycle. On one hand, reduced MMP acts as a critical initiating step that promotes PD‐1 upregulation on T cells.^33^ Concurrently, energy metabolism disruption resulting from mitochondrial dysfunction directly regulates PD‐1 gene transcription and expression.^30^ On the other hand, sustained PD‐1 signaling further maintains a low-MMP phenotype.^34^, ^35^ This vicious cycle ultimately impairs pulmonary immune defense and increases the risk of PPCs. Although mitochondrial metabolic abnormalities occupy a central role in this pathophysiological cycle, the present findings indicate that they are not independent predictors of PPCs. This may be attributable to the relatively advanced age of the enrolled patients. Mitochondrial function naturally declines with aging, and the generally low baseline mitochondrial function in this cohort may have, to some extent, masked its ability to discriminate PPCs related pathological abnormalities.^36^ Furthermore, the possibility that mitochondrial damage acts more as a downstream amplifier than an upstream initiator may further limit its independent predictive value.^37^, ^38^

Several limitations of this study should be acknowledged. First, as a single-center observational study, the findings require further validation in large-scale, multicenter prospective cohorts. Second, only preoperative immune indicators were assessed; the absence of dynamic postoperative measurements of T cell and mitochondrial function precludes comparison of immune function changes before and after surgery. Third, the limited sample size of the PPCs group precluded stratified subgroup analyses to thoroughly explore sources of heterogeneity. Fourth, detailed data on intraoperative anesthetic management (e.g., anesthetic agents, duration of one-lung ventilation) were not collected, which may have interacted with preoperative immune status and influence PPCs risk. The absence of these variables precluded adjustment for their potential confounding effects. Future prospective studies are expected to incorporate anesthetic data to address this limitation. Fifth, the generalizability of our prediction model requires external validation in larger independent cohorts, as the limited sample size precluded stable bootstrap-based internal validation. Finally, this study focused exclusively on T cell subsets. Expanding the detection scope to include innate immune cells such as neutrophils and monocytes—combined with a more comprehensive immune indicator analysis strategy—would allow a more systematic characterization of the perioperative immune microenvironment.

## Conclusions

In conclusion, the findings of this study provide preliminary evidence that an elevated percentage and absolute count of CD3^+^CD4^+^PD‐1^+^ T cells, along with prolonged operative time, are independent risk factors for PPCs in patients undergoing thoracic surgery, whereas a higher lymphocyte percentage is an independent protective factor. These results suggest that preoperative PD‐1 expression in T cell subsets may serve as a potential biomarker for assessing PPC risk in this patient population. Accordingly, perioperative immunomodulation, particularly early intervention based on preoperative immune status, may represent a promising preventive strategy to reduce the incidence of PPCs.

## CRediT authorship contribution statement

**Muhuo Ji**: Supervision, Conceptualization, Writing – review & editing. **Qingren Liu**: Supervision, Formal analysis, Writing – review & editing. **Jianjun Yang**: Supervision, Writing – review & editing. **Xingqi Zhao**: Data curation. **Tiantian Zhou**: Data curation. **Yuting Liu**: Formal analysis. **Wen Liu**: Writing – original draft. All authors have read and agreed to the published version of the manuscript.

## Consent for publication

Written informed consent has been obtained from the participants to publish this paper.

## Ethical statement

This study was reviewed and approved by the Ethics Committee of the Second Affiliated Hospital of Nanjing Medical University, Jiangsu Province, China (Approval No. 2024-KY−174–02).

## Funding

This research received no external funding.

## Declaration of competing interest

The authors declare that they have no known competing financial interests or personal relationships that could have appeared to influence the work reported in this paper. Jianjun Yang is the Editor-in-Chief of Journal of Anesthesia and Translational Medicine and was not involved in the editorial review or the decision to publish this article.

## Data Availability

The data that support the findings of this study are available from the corresponding author upon reasonable request.

## Footnotes

Appendix A Supplementary data associated with this article can be found in the online version at doi:10.1016/j.jatmed.2026.07.002.
