# Supplementary material for: Predictive value of preoperative T cell phenotypes for postoperative pulmonary complications in elderly patients undergoing thoracic surgery
Source: J Anesth Transl Med. 2026 Aug 10;5(3):162–70. doi: 10.1016/j.jatmed.2026.07.002 (PMC13485646; doi:10.1016/j.jatmed.2026.07.002)
Supplement: Supplementary file 1 [file mmc1.pdf]

Table S1. Comparison of preoperative T cell subset profiles between the two groups.

Variable	non-PPCs	PPCs	*P*-value
	N = 132	N = 44	
T cell subset percentages			
$CD3^+$ T cells (%)	71.17 (63.90,75.72)	68.66 (62.3,76.40)	0.643
$CD3^+CD4^+$ T cells (%)	43.73±8.76	40.80±8.57	0.055
$CD3^+CD8^+$ T cells (%)	20.37±7.79	22.53±10.18	0.143
T cell subset absolute counts			
$CD3^+$ T cells (cells/μL)	912.00 (702.00, 1285.75)	886.50 (596.00, 1155.25)	0.206
$CD3^+CD4^+$ T cells (cells/μL)	615.00 (431.25, 798.25)	511.50 (389.50, 668.25)	0.044
$CD3^+$CD8+ T cells (cells/μL)	245.00 (170.75, 398.00)	246.50 (187.25, 466.75)	0.929

Note: Data are presented as mean±standard deviation or median (interquartile range) depending on variable type and distribution. PPCs, postoperative pulmonary complications. P<0.05 was considered statistically significant.

Table S2-1. Comparison of preoperative PD-1 expression in T cell subsets between the two groups.

Variable	non-PPCs N = 132	PPCs N = 44	*P*-value
$CD3^+CD4^+PD\text{-}1^+$ T cells (%)	17.65±6.34	24.35±8.44	<0.001
$CD3^+CD8^+PD\text{-}1^+$ T cells (%)	22.78 (17.70, 31.37)	26.46 (20.53, 33.55)	0.105
$CD3^+CD4^+PD\text{-}1^+$ T cells (cells/μL)	100.50 (82.25, 125.75)	127.50 (80.75, 183.00)	0.029
$CD3^+CD8^+PD\text{-}1^+$ T cells (cells/μL)	59.50 (43.00, 88.75)	72.00 (48.00, 102.50)	0.150

Note: Data are presented as mean±standard deviation or median (interquartile range) depending on variable type and distribution. PD-1, programmed cell death protein-1; PPCs, postoperative pulmonary complications. *P*<0.05 was considered statistically significant.

Table S2-2. Comparison of preoperative mitochondrial function-related indicators in T cell subsets between the two groups.

Variable	non-PPCs N = 132	PPCs N = 44	*P*-value
$CD3^+$MMP T cells （%）	18.84 (15.36, 25.17)	18.52 (14.33, 21.67)	0.096
$CD3^+CD4^+$MMP T cells （%）	15.55 (11.79, 20.63)	14.27 (11.41, 18.41)	0.119
$CD3^+CD8^+$MMP T cells （%）	25.17 (19.04, 32.16)	21.69 (17.05, 24.49)	0.010
$CD3^+$T cell MM	2.63 (2.16, 3.58)	2.85 (2.24, 3.76)	0.319
$CD3^+CD4^+$T cell MM	2.76 (2.27, 3.71)	3.06 (2.41, 3.80)	0.339
$CD3^+CD8^+$T cell MM	2.46 (2.05, 3.48)	2.67 (2.17, 3.75)	0.212

Note: Data are presented as mean ± standard deviation or median (interquartile range) depending on variable type and distribution. MMP, mitochondrial membrane potential; MM, mitochondrial mass; PPCs, postoperative pulmonary complications. *P*<0.05 was considered statistically significant.

Table S3-1. Univariate logistic regression analysis of risk factors for PPCs.

	OR	95% CI	*P*-value
Age	1.072	1.012-1.136	0.017
Operation Time	1.005	1.002-1.009	0.004
Pre Neutrophil Count	1.106	0.886-1.379	0.373
Lym Percentage	0.960	0.925-0.997	0.034
WBC	1.019	0.875-1.185	0.813
CRP	1.023	0.992-1.056	0.148
D-Dimer	2.038	1.051-3.954	0.035
FDP	1.078	0.940-1.237	0.282
PT	1.224	0.937-1.600	0.138
APTT	1.040	0.986-1.098	0.150
INR	0.897	0.583-1.381	0.622
$CD3^+CD4^+PD\text{-}1^+$ T cell percentage	1.132	1.075-1.193	<0.001
$CD3^+CD4^+$ T cells count	0.999	0.997-1.000	0.067
$CD3^+CD4^+PD\text{-}1^+$ T cells count	1.009	1.003-1.015	0.006
$CD3^+CD8^+$MMP T cell percentage	0.948	0.910-0.988	0.011

Note: Lym, lymphocyte; WBC, white blood cell; CRP, C-reactive Protein; PT, Prothrombin Time; APTT, Activated Partial Thromboplastin Time; FDP, Fibrinogen Degradation Products; INR, international normalized ratio; PD-1, programmed cell death protein-1; MMP, mitochondrial membrane potential; PPCs, postoperative pulmonary complications; OR, odds ratio; CI, confidence interval. $P<0.05$ was considered statistically significant.

Table S3-2. Multivariable logistic regression analysis of $CD3^+CD4^+PD\text{-}1^+$ T cell percentage for PPCs.

	OR	95% CI	*P*-value
Age	1.030	0.960-1.104	0.412
Operation Time	1.008	1.004-1.013	0.001
Lym Percentage	0.988	0.940-1.038	0.625
WBC	0.952	0.717-1.264	0.734
CRP	1.013	0.968-1.061	0.575
D-Dimer	3.365	0.456-24.845	0.234
FDP	0.730	0.379-1.405	0.346
PT	1.050	0.646-1.708	0.844
APTT	0.980	0.900-1.066	0.634
INR	0.752	0.235-2.409	0.752
$CD3^+CD4^+PD\text{-}1^+$ T cell percentage	1.140	1.074-1.210	<0.001
$CD3^+CD8^+$MMP T cell percentage	0.955	0.908-1.004	0.073

Note: Lym, lymphocyte; WBC, white blood cell; CRP, C-reactive Protein; PT, Prothrombin Time; APTT, Activated Partial Thromboplastin Time; FDP, Fibrinogen Degradation Products; INR, international normalized ratio; PD-1, programmed cell death protein-1; MMP, mitochondrial membrane potential; PPCs, postoperative pulmonary complications; OR, odds ratio; CI, confidence interval. $P<0.05$ was considered statistically significant.

Table S3-3. Multivariable logistic regression analysis of absolute count of $CD3^{+}CD4^{+}PD\text{-}1^{+}$ T cells for PPCs.

	OR	95% CI	*P*-value
Age	1.040	0.971-1.114	0.260
Operation Time	1.007	1.003-1.012	0.002
Lym Percentage	0.926	0.873-0.982	0.010
WBC	0.707	0.464-1.078	0.107
CRP	1.022	0.974-1.073	0.379
D-Dimer	3.516	0.592-20.900	0.167
FDP	0.805	0.456-1.421	0.454
PT	1.139	0.697-1.861	0.603
APTT	0.972	0.892-1.060	0.523
INR	0.724	0.212-2.480	0.608
$CD3^{+}CD4^{+}PD\text{-}1^{+}$ T cells count	1.020	1.011-1.030	<0.001
$CD3^{+}CD8^{+}$MMP T cell percentage	0.952	0.905-1.001	0.053

Note: Lym, lymphocyte; WBC, white blood cell; CRP, C-reactive Protein; PT, Prothrombin Time; APTT, Activated Partial Thromboplastin Time; FDP, Fibrinogen Degradation Products; INR, international normalized ratio; PD-1, programmed cell death protein-1; MMP, mitochondrial membrane potential; PPCs, postoperative pulmonary complications; OR, odds ratio; CI, confidence interval. $P<0.05$ was considered statistically significant.

Table S4-1. Full multivariable model with $CD3^+CD4^+PD\text{-}1^+$ T cell percentage and all covariates.

**Model 2**	**OR**	**95% CI**	***P*-value**
Age	1.057	0.990-1.127	0.095
Operation Time	1.007	1.003-1.012	0.001
$CD3^+CD4^+PD\text{-}1^+$ T cell percentage	1.151	1.086-1.219	<0.001
**Model 3**			
Age	1.034	0.967-1.106	0.327
Operation Time	1.008	1.003-1.037	0.001
Lym Percentage	0.991	0.947-1.037	0.699
D-Dimer	1.443	0.732-2.846	0.290
$CD3^+CD8^+$MMP T cell percentage	0.952	0.907-0.999	0.043
$CD3^+CD4^+PD\text{-}1^+$ T cell percentage	1.137	1.072-1.205	<0.001

Table S4-2. Full multivariable model with $CD3^+CD4^+PD\text{-}1^+$ T cells count and all covariates.

**Model 2**	**OR**	**95% CI**	***P*-value**
Age	1.080	1.014-1.151	0.017
Operation Time	1.006	1.002-1.010	0.002
CD3+CD4+PD-1+ T cells count	1.012	1.005-1.018	0.001
**Model 3**			
Age	1.055	0.988-1.126	0.112
Operation Time	1.007	1.003-1.011	0.002
Lym Percentage	0.965	0.922-1.010	0.122
D-Dimer	1.701	0.843-3.431	0.138
$CD3^+CD8^+$MMP T cell percentage	0.952	0.908-0.997	0.038
$CD3^+CD4^+PD\text{-}1^+$ T cells count	1.013	1.006-1.021	<0.001

Table S5-1. ROC curve of $CD3^+CD4^+PD\text{-}1^+$ T cell percentage for PPCs.

	AUC (95% CI)	Sensitivity (%)	Specificity (%)	Cutoff value	*P*-value
$CD3^+CD4^+PD\text{-}1^+$ T cell percentage	0.741 (0.649-0.834)	59.1	87.1	23.045	<0.001
Operation Time	0.627 (0.529-0.725)	63.6	60.6	122.5	0.012
Combined test 1	0.815 (0.749-0.869)	59.1	89.4		<0.001

Note: Combined test 1 refers to the combination of $CD3^+CD4^+PD\text{-}1^+$ T cell percentage and operative time for predicting PPCs. PD-1, programmed cell death protein-1; AUC, area under the ROC curve; CI, confidence interval. $P<0.05$ was considered statistically significant.

Table S5-2. ROC curve of absolute count of $CD3^+CD4^+PD\text{-}1^+$ T cells for PPCs.

	AUC (95% CI)	Sensitivity (%)	Specificity (%)	Cutoff value	*P*-value
$CD3^+CD4^+PD\text{-}1^+$ T cells count	0.610 (0.500-0.721)	52.3	76.5	126.5	0.029
Operation Time	0.627 (0.529-0.725)	63.6	60.6	122.5	0.012
Lym Percentage	0.610 (0.514-0.705)	51.5	70.5	32.1	0.030
Combined test 2	0.777 (0.708-0.836)	77.3	65.2		<0.001

Note: Combined test 2 refers to the combination of absolute count of $CD3^+CD4^+PD\text{-}1^+$ T cells, operative time, and lymphocyte percentage for predicting PPCs. Lym, lymphocyte; PD-1, programmed cell death protein-1; AUC, area under the ROC curve; CI, confidence interval. $P<0.05$ was considered statistically significant.

Table S6-1. Sensitivity analysis for non-linearity using quadratic term in the full multivariable logistic regression model ($CD3^+CD4^+PD\text{-}1^+$ T cell percentage).

Model	AIC	Residual deviance	df	Quadratic term P value	Likelihood ratio test P value
Linear model	164.11	150.11	169	\	\
Quadratic model	166.09	150.09	168	0.890	0.890

Note: Both models were adjusted for all covariates that reached statistical significance in univariate analysis ($P<0.05$), including age, operative time, lymphocyte percentage, D-dimer, $CD3^+CD4^+PD\text{-}1^+$ T cell percentage, and $CD3^+CD8^+$MMP T cell percentage. The quadr atic term was entered as ($CD3^+CD4^+PD\text{-}1^+$ T cell percentage)² to formally test for non-linearity. The non-significant quadratic term (P =0.890) and the non-significant likelihood ratio test (P=0.890) confirm that a linear model provides an adequate fit for the data, supp orting the linear dose-response relationship reported in the main analysis. AIC, Akaike Information Criterion; df, degrees of freedom.

Table S6-2. Sensitivity analysis for non-linearity using quadratic term in the full multivariable logistic regression model ($CD3^+CD4^+PD\text{-}1^+$ T cells count).

Model	AIC	Residual deviance	df	Quadratic term P value	Likelihood ratio test P value
Linear model	173.10	159.10	169	\	\
Quadratic model	174.45	158.45	168	0.430	0.419

Note: Both models were adjusted for all covariates that reached statistical significance in univariate analysis ($P<0.05$), including age, operative time, lymphocyte percentage, D-dimer, $CD3^+CD4^+PD\text{-}1^+$ T cells count, and $CD3^+CD8^+$MMP T cell percentage. The quadratic term was entered as ($CD3^+CD4^+PD\text{-}1^+$ T cells count)² to formally test for non-linearity. The non-significant quadratic term (P=0.430) and the non-significant likelihood ratio test (P=0.419, ΔAIC = +1.35) confirm that a linear model provides an adequate fit for the d ata, supporting the linear dose-response relationship reported in the main analysis. AIC, Akaike Information Criterion; df, degrees of f reedom.

Table S7-1. Spearman correlation between CD3⁺CD4⁺PD-1⁺ T cell percentage and count.

	**$CD3^+CD4^+PD\text{-}1^+$ T cell percentage**	**$CD3^+CD4^+PD\text{-}1^+$ T cells count**
$CD3^+CD4^+PD\text{-}1^+$ T cell percentage	1.000	0.387
$CD3^+CD4^+PD\text{-}1^+$ T cells count	0.387	1.000

Table S7-2. Spearman correlation matrix for $CD3^+CD4^+PD\text{-}1^+$ T cell percentage.

	**1**	**2**	**3**	**4**	**5**	**6**	**7**	**8**	**9**	**10**	**11**	**12**
$CD3^+CD4^+PD\text{-}1^+$ T cell percentage	1.000											
age	0.108	1.000										
Operation Time	-0.106	0.144	1.000									
Lym Percentage	-0.202**	-0.205**	-0.030	1.000								
WBC	0.046	0.047	-0.023	-0.362**	1.000							
CRP	0.134	0.127	0.113	-0.364**	0.210**	1.000						
D-Dimer	0.107	0.253**	0.076	-0.317**	0.124	0.276**	1.000					
FDP	0.090	0.259**	0.171*	-0.327**	0.019	0.243**	0.748	1.000				
TT	0.060	0.101	-0.200**	0.102	-0.158*	-0.298**	-0.113	-0.185*	1.000			
APTT	0.239**	0.059	0.018	-0.074	0.067	0.229**	0.117	0.015	-0.027	1.000		
INR	0.131	0.117	0.197**	-0.175*	-0.083	0.224**	0.123	0.190*	0.063	0.243**	1.000	
$CD3^+CD8^+$MMP T cell percentage	-0.264**	-0.242**	0.137	0.131	-0.116	-0.024	-0.170*	-0.054	-0.027	-0.147	-0.006	1.000

Note: All correlation coefficients are Spearman's ρ. *, $P<0.05$; **, $P<0.01$ (two-tailed). Bold values indicate correlation coefficients ≥ 0.5. WBC, white blood cell count; CRP, C-reactive protein; FDP, fibrinogen degradation products; TT, thrombin time; APTT, activated partial thromboplastin time; INR, international normalized ratio.

Table S7-3. Spearman correlation matrix for CD3$^+$CD4$^+$PD-1$^+$ T cells count.

	1	2	3	4	5	6	7	8	9	10	11	12
CD3$^+$CD4$^+$PD-1$^+$ T cells count	1.000											
age	-0.159*	1.000										
Operation Time	-0.056	0.144	1.000									
Lym Percentage	0.264**	-0.205**	-0.030	1.000								
WBC	0.394**	0.047	-0.023	-0.362**	1.000							
CRP	-0.015	0.127	0.113	-0.364**	0.210**	1.000						
D-Dimer	-0.178*	0.253**	0.076	-0.317**	0.124	0.276**	1.000					
FDP	-0.261**	0.259**	0.171*	-0.327**	0.019	0.243**	0.748	1.000				
TT	0.027	0.101	-0.200**	0.102	-0.158*	-0.298**	-0.113	-0.185*	1.000			
APTT	0.060	0.059	0.018	-0.074	0.067	0.229**	0.117	0.015	-0.027	1.000		
INR	-0.054	0.117	0.197**	-0.175*	-0.083	0.224**	0.123	0.190*	0.063	0.243**	1.000	
CD3$^+$CD8$^+$MMP T cell percentage	-0.090	-0.242**	0.137	0.131	-0.116	-0.024	-0.170*	-0.054	-0.027	-0.147	-0.006	1.000

Note: All correlation coefficients are Spearman's ρ. *, P<0.05; **, P<0.01 (two-tailed). Bold values indicate correlation coefficients ≥ 0.5. WBC, white blood cell count; CRP, C-reactive protein; FDP, fibrinogen degradation products; TT, thrombin time; APTT, activated partial thromboplastin time; INR, international normalized ratio.

Table S8. Model performance and calibration assessment of the model 3.

	AUC	Brier score	HL χ^2	HL df	HL *P* value
CD3$^+$CD4$^+$PD-1$^+$ T cell percentage	0.815	0.137	7.605	8	0.473
CD3$^+$CD4$^+$PD-1$^+$ T cell absolute count	0.777	0.137	3.497	8	0.899

Note: AUC, area under the receiver operating characteristic curve. Brier score < 0.25 indicates acceptable overall performance. A non-significant Hosmer-Lemeshow test (P>0.05) supports adequate calibration for both models.

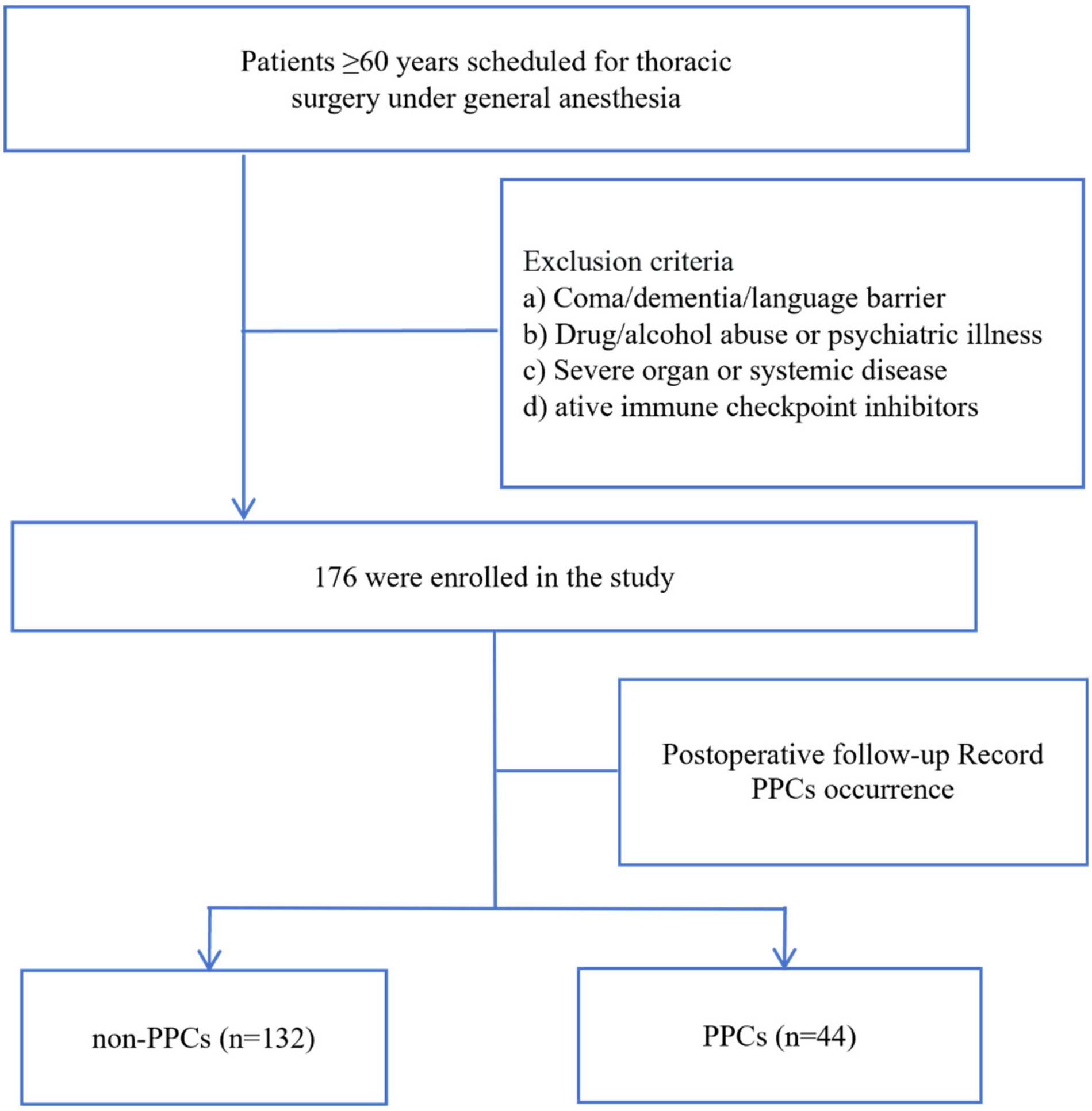

Figure S1. Study flow chart.

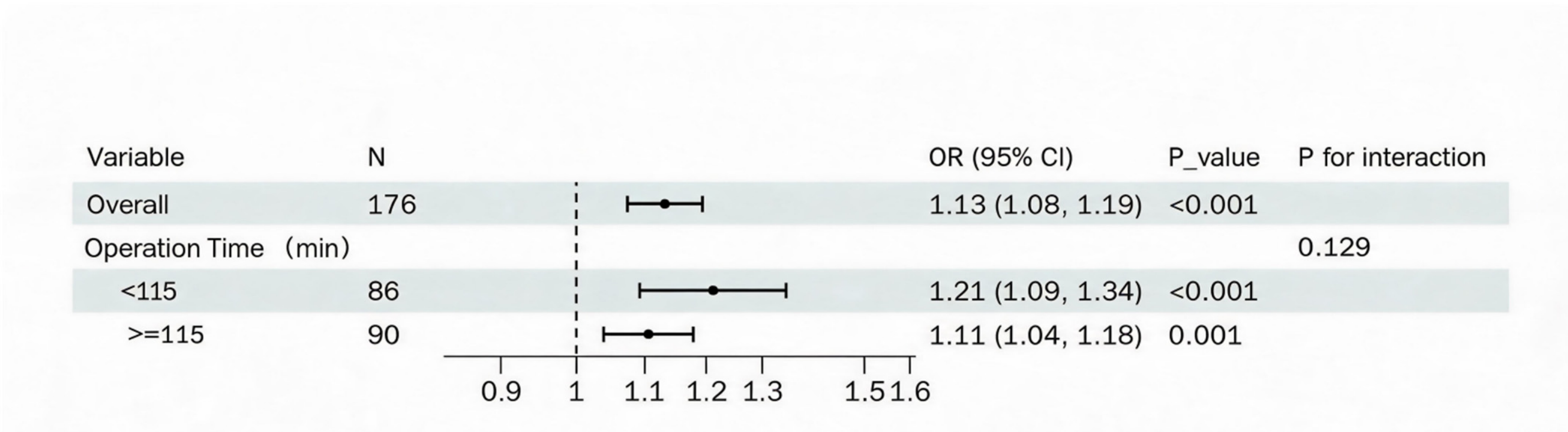

Figure S2. Subgroup analysis of $CD3^{+}CD4^{+}PD\text{-}1^{+}$ T cell percentage and PPCs by operative time.

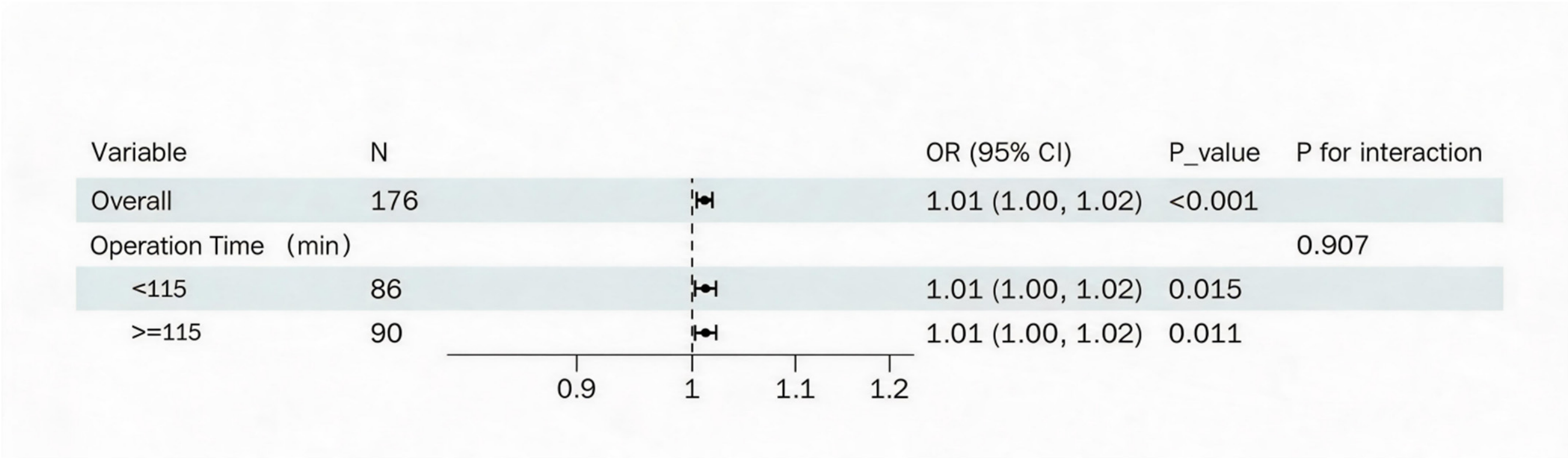

Figure S3. Subgroup analysis of $CD3^{+}CD4^{+}PD\text{-}1^{+}$ T cells count and PPCs by operative time.

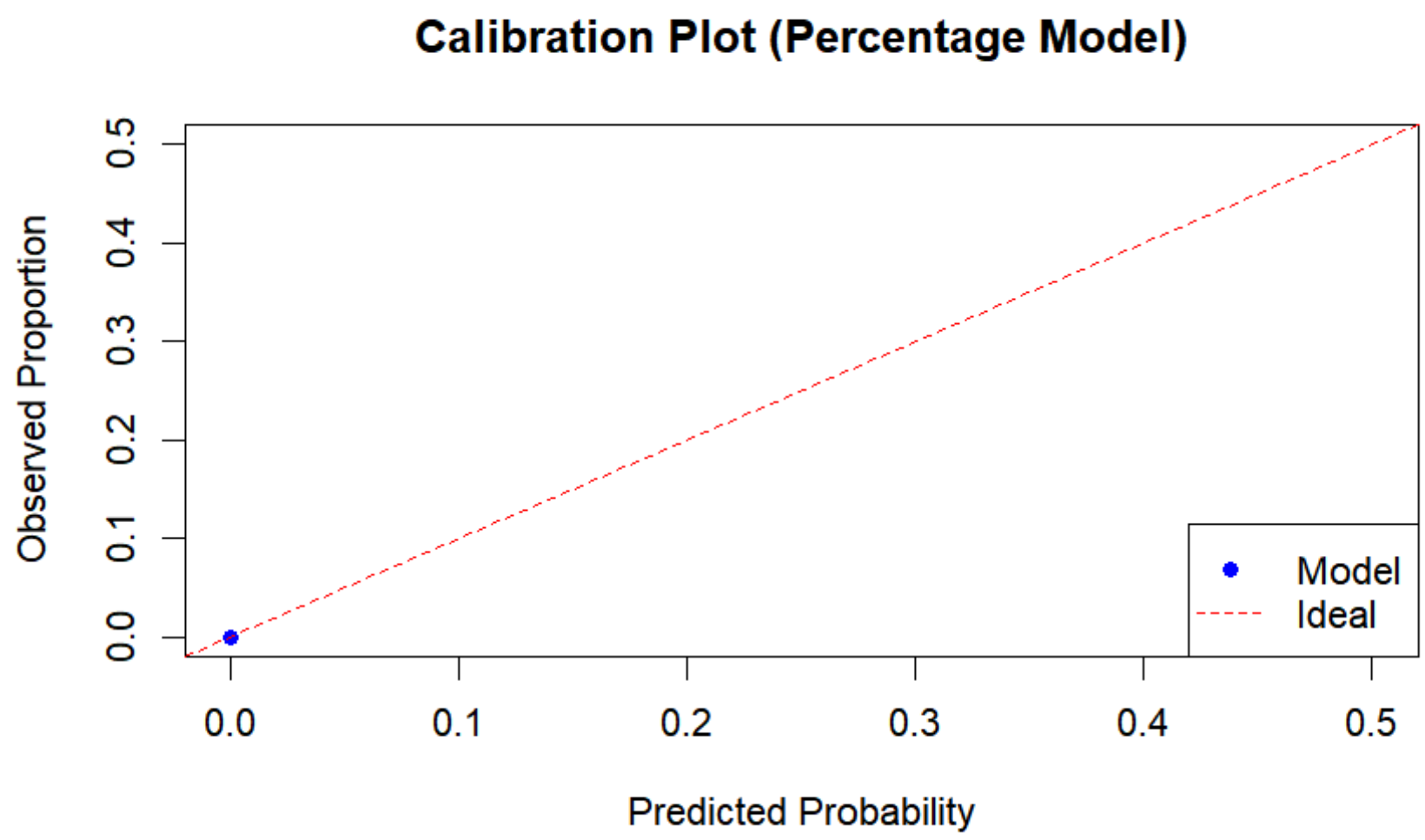

Figure S4-1. Calibration plots for Model 3 ($CD3^{+}CD4^{+}PD\text{-}1^{+}$ T cell percentage).

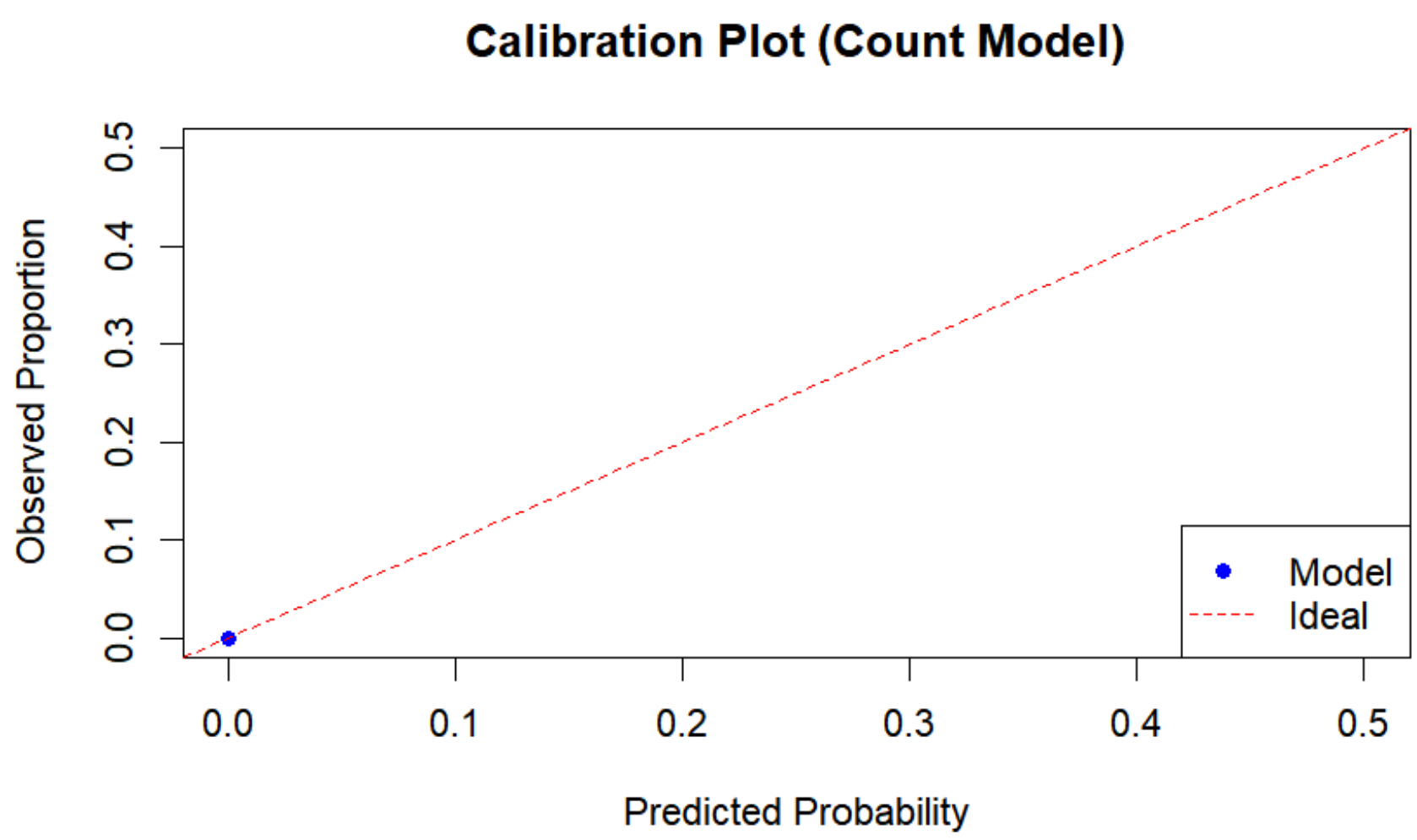

Figure S4-2. Calibration plots for Model 3 ($CD3^{+}CD4^{+}PD\text{-}1^{+}$ T cells count).
