# Supplementary material for: Predictive value of preoperative T cell phenotypes for postoperative pulmonary complications in elderly patients undergoing thoracic surgery
Source: J Anesth Transl Med. 2026 Aug 10;5(3):162–70. doi: 10.1016/j.jatmed.2026.07.002 (PMC13485646; doi:10.1016/j.jatmed.2026.07.002)

Flow Cytometry Methodology

1, Lymphocytes were gated on an FSC-A vs SSC-A density plot

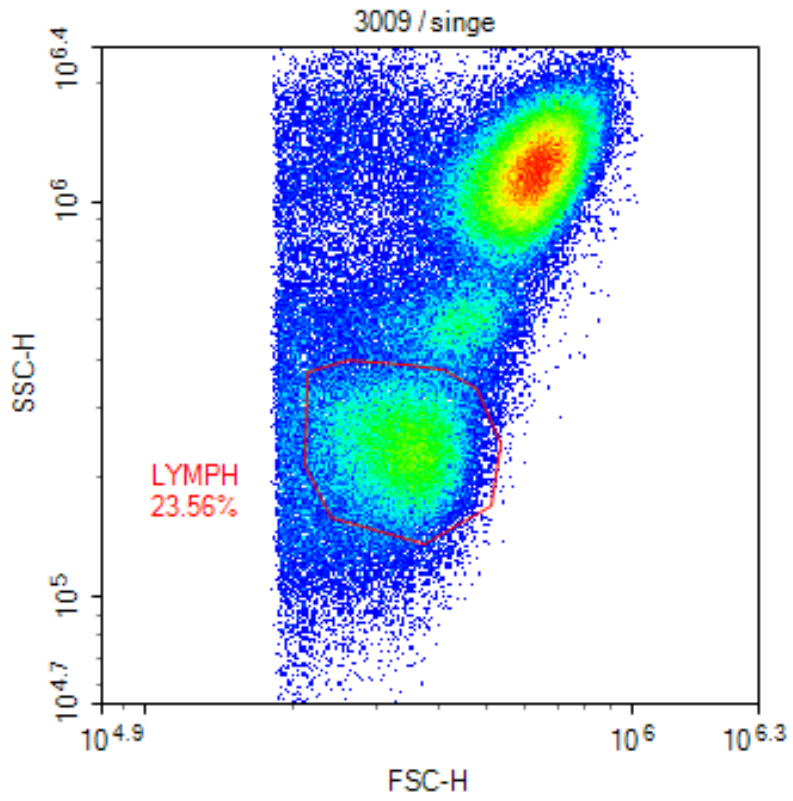

2, From the lymphocyte gate, create a CD4/CD8 density plot and gate the CD4+CD8- and CD4-CD8+ cell populations, as shown in the figure

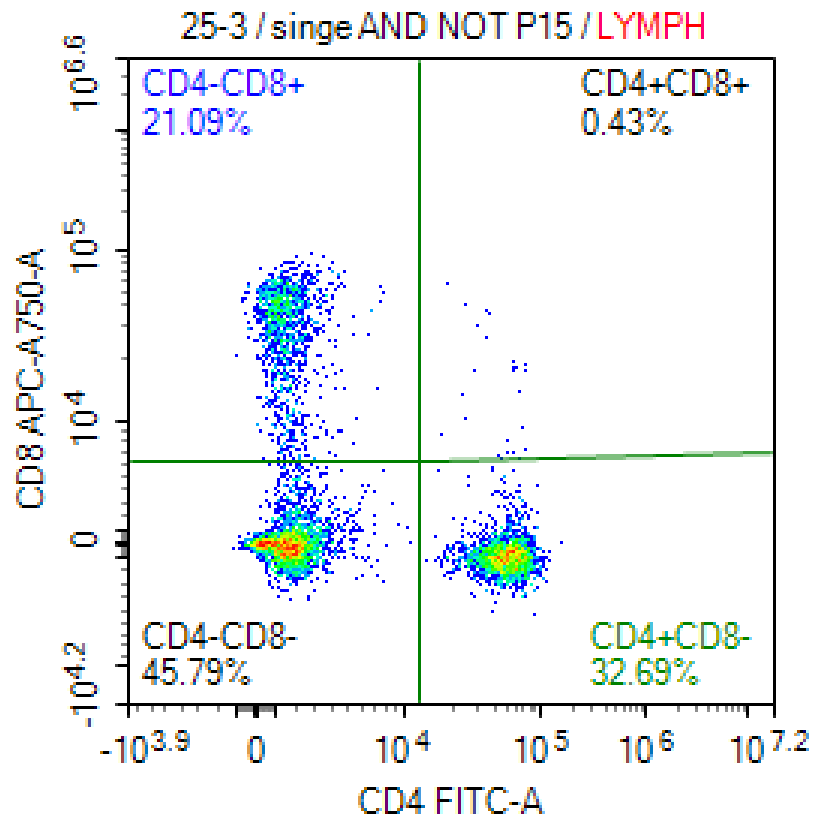

3, Gate on CD4+CD8- cells, generate a CD62L vs CD45RA density plot, draw a quadrant gate, and identify T4Tn, T4Tcm, T4Tem, and T4Tef cell populations

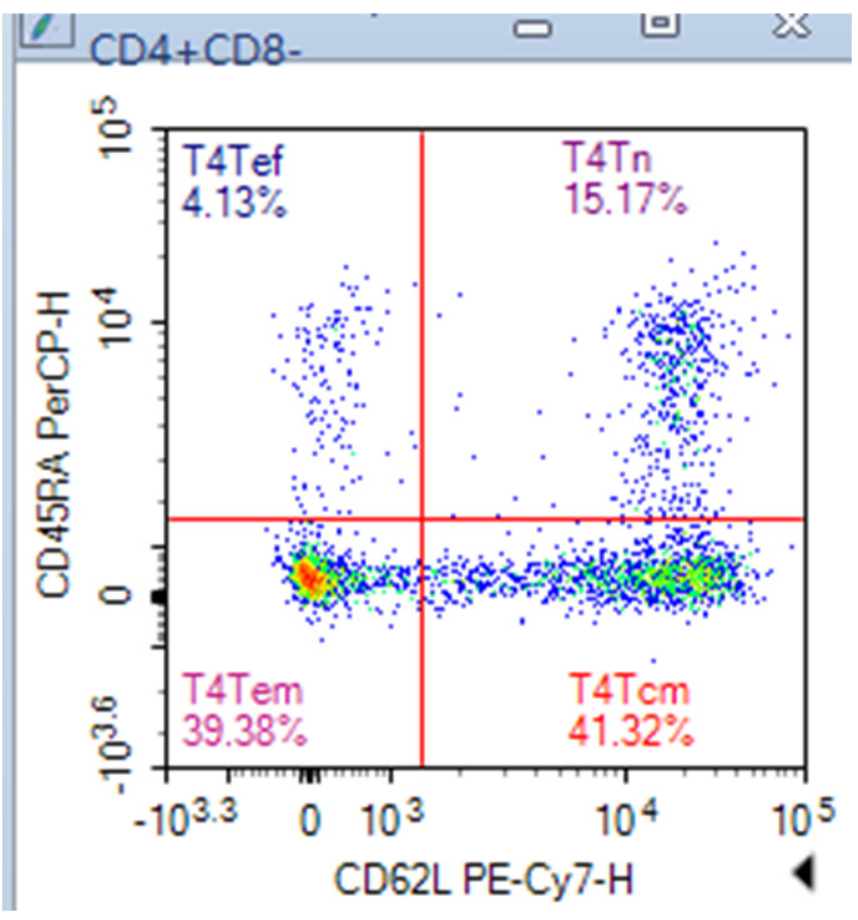

4, Gate on CD4-CD8+ cells, generate a CD62L vs CD45RA density plot, draw a quadrant ga te, and identify T8Tn, T8Tcm, T8Tem, and T8Tef cell populations

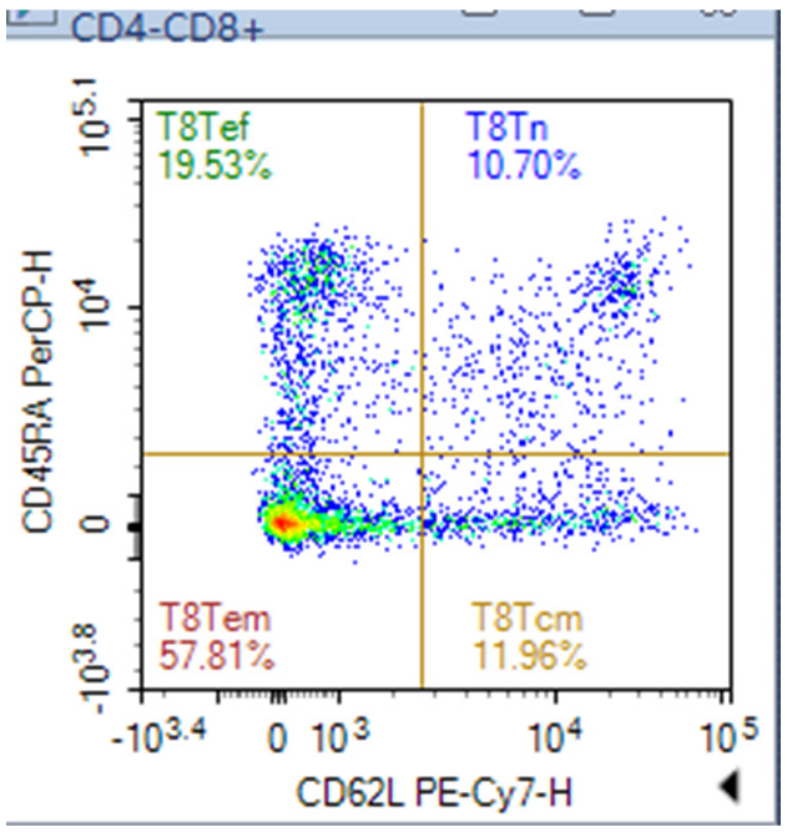

5, Using region gates, the percentage of MITO APC-H^+ cells within the Tef and Tem subsets of $CD4^+CD8^-$ (T4) and $CD4^-CD8^+$ (T8) populations, as well as the percentage of each subs et among total lymphocytes and the fluorescence intensity, were calculated

T4:

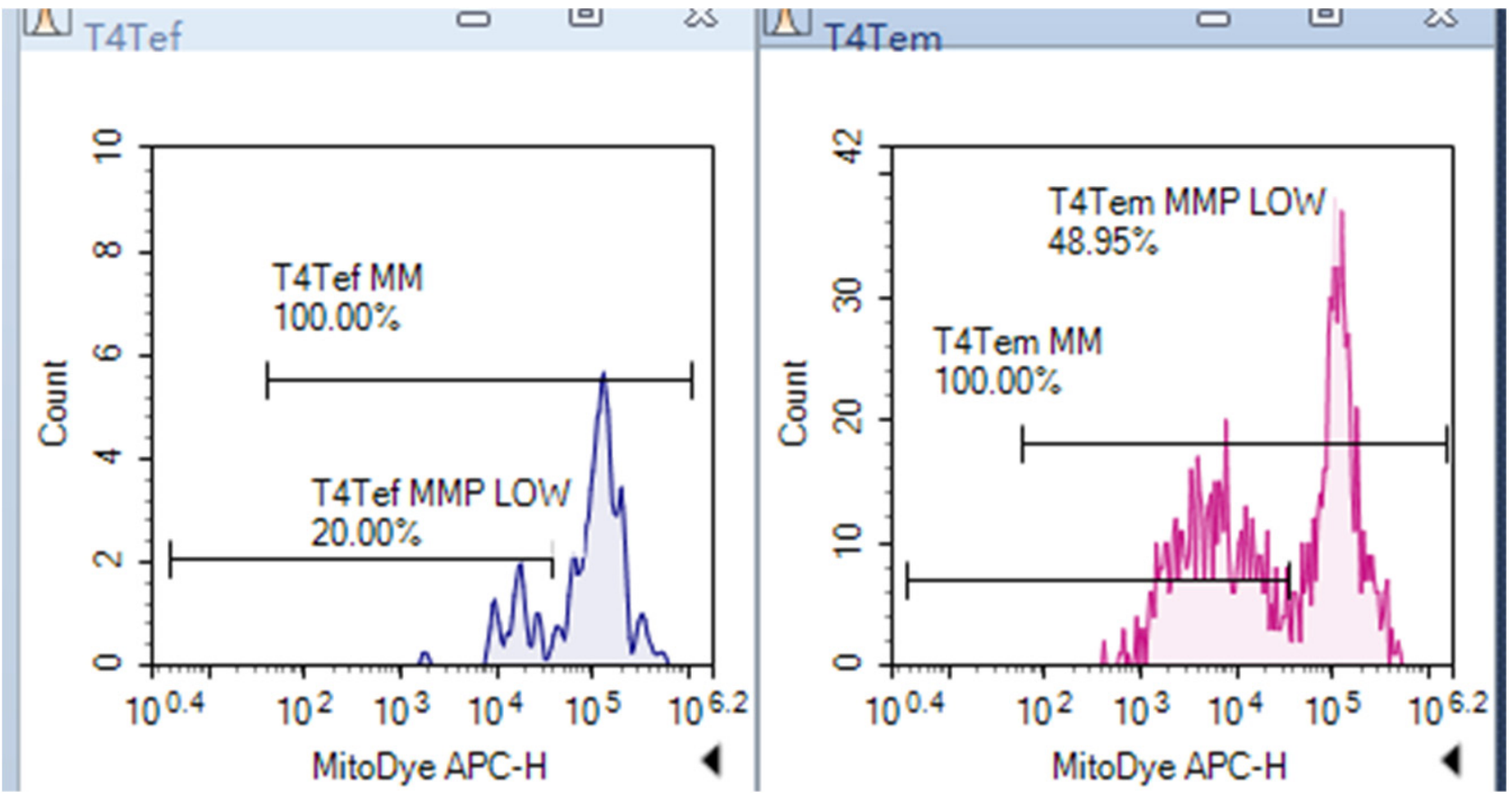

T8:

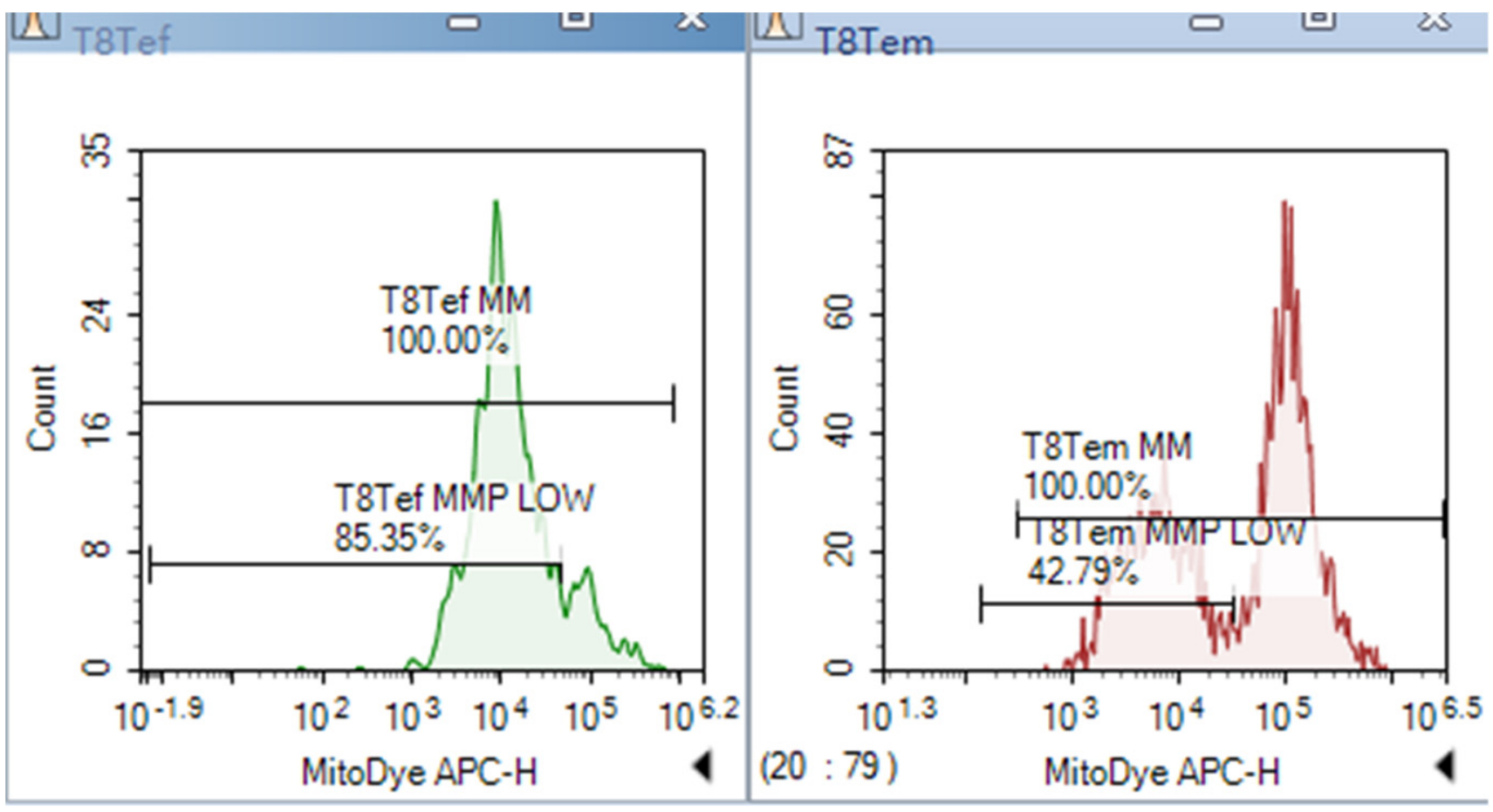

6, Using rectangular region gates, $CD4^+$, $CD8^+$, T4Tef, T4Tem, T8Tef, and T8Tem cell popula tions were gated, and the percentage of $PD\text{-}1^+$ cells within each population was calculated

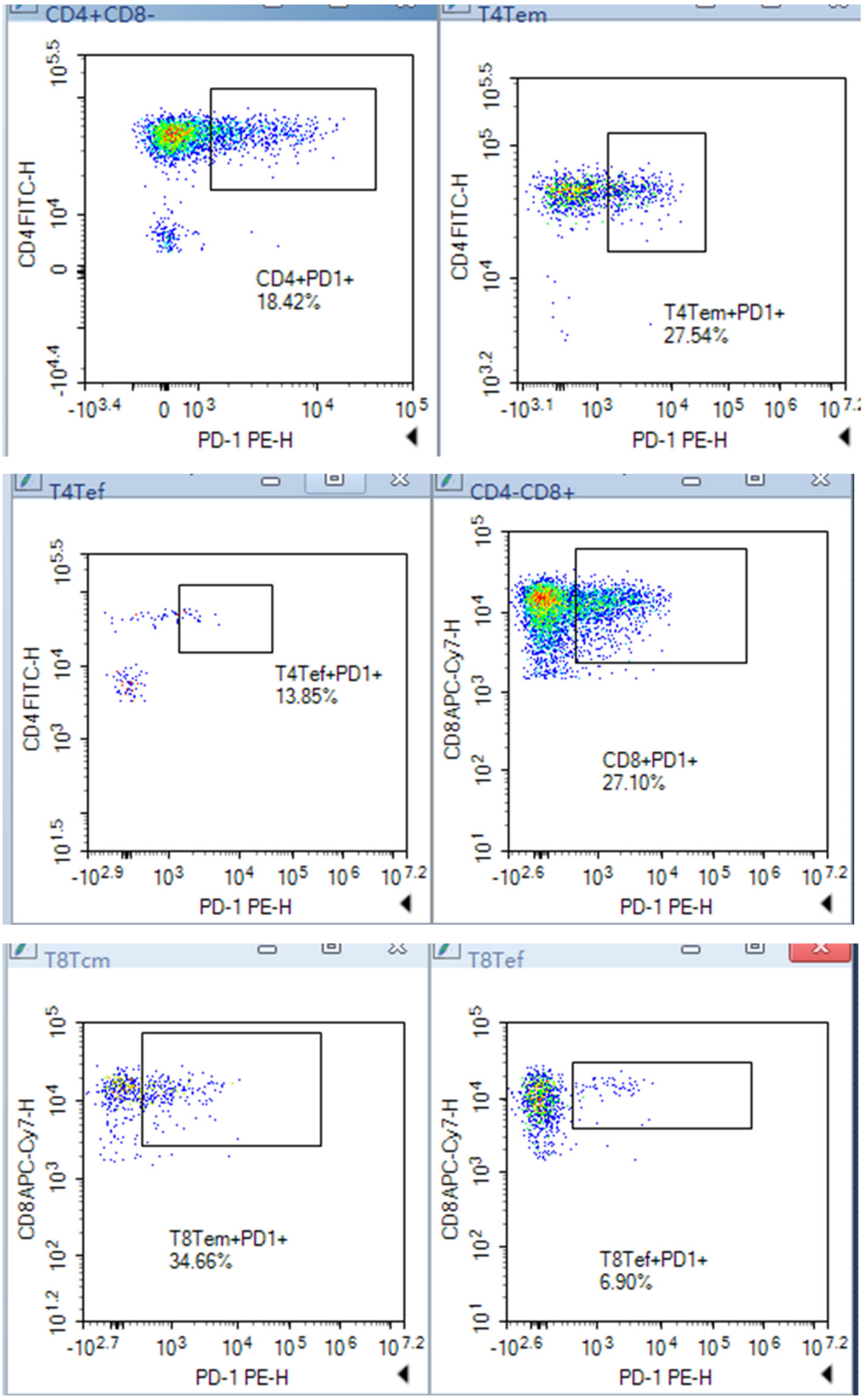

Supplement: Supplementary file 2 [file mmc2.pdf]
